# Saffron as a Retinal Neuroprotectant: A Narrative Review of Preclinical Studies and Clinical Results

**DOI:** 10.3390/antiox15040501

**Published:** 2026-04-17

**Authors:** Maria Anna Maggi, Rocco Mastromartino, Marco Piccardi, Angelo Maria Minnella, Dario Marangoni, Stefano Di Marco, Benedetto Falsini, Silvia Bisti

**Affiliations:** 1 Hortus Novus Srl, Via Campo Sportivo 2, 67050 Canistro, Italy; m.maggi@hortusnovus.it; 2 Dipartimento di Scienze Della Vita e Sanità Pubblica, Sezione di Medicina Genomica, Università Cattolica del Sacro Cuore, 00168 Rome, Italy; rocco.mastromartino01@icatt.it; 3 Dipartimento di Scienze Otorinolaringoiatriche ed Oftalmologiche, Università Cattolica del Sacro Cuore, 00168 Rome, Italy; marco.piccardi2@unicatt.it (M.P.); angelomaria.minnella@unicatt.it (A.M.M.); 4 Dipartimento Universitario Clinico di Scienze Mediche, Chirurgiche e Della Salute, Clinica Oculistica, Università di Trieste, 34151 Trieste, Italy; dariomarangoni80@gmail.com; 5 Center for Synaptic Neuroscience (NSYN), Italian Institute of Technology (IIT), 16152 Genoa, Italy; stefano.dimarco@unige.it; 6 Dipartimento di Medicina Sperimentale, DIMES Unige, 16132 Genova, Italy; 7 Istituto Nazionale Biosistemi e Biostrutture (INBB), 00136 Roma, Italy; s.bisti@team.it

**Keywords:** saffron, crocins, retina, neurodegeneration, visual function, oxidative stress, inflammation

## Abstract

The present narrative review reports the main preclinical and clinical results obtained by using supplementation of saffron or its pure components in neurodegeneration, with special emphasis on age-related macular degeneration. Beyond that, this article will address shared pathways between neurodegenerative diseases of the eye and the brain. It will be shown that saffron treatment might counteract oxidative damage in the retina and brain, as well as inflammation and inflammatory mediators that induce neuronal degeneration and death. The ways of action are multiple, and saffron chemical components appear to act in a synergistic manner, inducing tissue resilience. These effects critically depend upon the saffron chemical composition and structure. A well-defined ratio among molecules is linked to a patented batch known as Repron^®^ and offers the maximum protection against neurodegeneration.

## 1. Introduction

Neurodegeneration represents a shared pathobiological substrate across numerous nervous system diseases, such as Amyotrophic Lateral Sclerosis, Alzheimer’s disease, Parkinson’s disease, head trauma, epilepsy and stroke. These disorders impose a substantial clinical and socioeconomic burden. In Europe, by 2040, patients affected by age-related macular degeneration (AMD) will be between 14.9 and 21.5 million [1]. Globally, the projected number will increase from 196 million to 288 million from 2020 to 2040 [2]. In the USA, annual costs are currently exceeding several hundred billion dollars, and current treatments are inadequate. Adding to the urgency of the problem is the fact that the incidence of these age-related disorders is increasing rapidly as population demographics change. Among nervous system disorders, there are specific neurodegenerative diseases of the neuro-retina of the eye. The visual system is the sensory modality that enables living organisms to explore the surrounding visual environment. Thus, the quality of vision is tightly linked to the quality of life. This link has probably motivated extensive basic and clinical research throughout the centuries, converting the visual system into one of the best instruments to understand the general principles of neurodevelopment, neurodegeneration and repair. As a result, there is already a wealth of information on how the visual system is built and how connections from the eye to the cortex enable visual perception. Despite this knowledge, many diseases that impair vision, ranging from Retinitis Pigmentosa (RP) to AMD, lack effective treatments.

AMD is a retinal neurodegenerative disease characterized in its early stage by large soft drusen and hypo-hyperpigmentation of the retinal pigment epithelium (RPE). Late AMD, the potentially blinding stage of disease, includes geographic atrophy of the RPE (“dry” AMD), or subretinal neovascular membranes (“wet” AMD) [3].

AMD is considered a multifaceted disease, whose development and progression are the result of a complex interaction between genetic and environmental risk factors. Both oxidative stress and chronic inflammation [4,5] seem to play a significant role in the pathogenesis of AMD, together with many other risk factors [6,7,8,9]. The outcome of all neurodegenerative retinal diseases is the death of photoreceptors; consequently, visual functions progressively deteriorate towards complete blindness. Recently, new strategies able to mitigate photoreceptor death using natural products have been explored [10,11,12,13]. Among the others, Maccarone et al. [14] provided data showing that saffron is protective against light-induced damage to photoreceptors in a rat model. Early anecdotal clinical results suggested potential efficacy in human patients. In an open pilot study of an AMD patient [15], focal-ERG increased in amplitude over a 3-month period, and focal-ERG phase and visual acuity were stable.

A proof-of-principle, randomized, placebo-controlled clinical trial in AMD patients corroborated the therapeutic potential of saffron treatment in neurodegenerative diseases and its consistency over time [15,16] and in patients carrying genetic variants predisposing to AMD [9].

Saffron is a well-known spice, used widely in traditional medical practice [17,18]. In more recent times, it has been used as an anti-inflammatory, anticonvulsive and anti-tumor agent; it has been explored in the treatment of cognitive defects in in vivo and in vitro models. Given its broad historical use and heterogeneous clinical applications, rigorous, indication-specific analyses are essential in retinal diseases. Saffron is the commercial name of the dried red stigmas of *Crocus sativus* L. flowers. It is produced in many areas all over the world, the bulbs do not present major different genetical characteristics (it is a sterile triploid species), but the cultivar and the drying strategies are quite different; this makes every single production unique, and this could explain the variety of effects and some discrepancies in the literature [19].

The chemical composition of saffron is highly complex and plays a critical role in determining its various biological properties. Studies conducted on animal models quickly revealed that not all saffron batches exert the same degree of neuroprotective efficacy [20,21]. These initial observations suggested that variability in chemical composition could be a key factor influencing bioactivity. However, it was only through a rigorous and integrated approach—combining in vitro cellular assays, in vivo pharmacological evaluations, and comprehensive chemical profiling—that the molecular basis for this variability was elucidated. This multidisciplinary strategy enabled researchers to identify significant differences in the metabolite composition of various saffron samples, ultimately demonstrating that only those with a specific chemical profile exhibited substantial neuroprotective effects. As reported by Di Marco et al. [20] and Maggi et al. [21], the therapeutic potential of saffron is closely linked to the concentration of a defined group of bioactive compounds, particularly crocins. Based on these findings, a specific metabolite composition was recognized as essential for consistent pharmacological efficacy. The chemical standardization of saffron-derived preparations is therefore of great importance, and the implementation of analytical certification tools such as Repron^®^ is strongly supported to distinguish pharmacologically active products from substandard ones.

## 2. Correlation Between Saffron Chemical Composition and Neuroprotective Efficacy

A rigorous metabolomic characterization of *Crocus sativus* L. (Iridaceae) is essential to elucidate its potential neuroprotective effects. Commonly referred to as “saffron”, this term is frequently misused, often applied indiscriminately to plant material that does not meet botanical or chemical criteria. True saffron is derived from the dried stigmas of the *Crocus sativus* flower, a highly aromatic plant recognized for its multifaceted phytochemical profile.

The floral structure of *Crocus sativus* comprises several distinct anatomical components, each contributing differently to the plant’s biochemical profile. Each flower contains three stigmas—highly valued due to their rich secondary metabolite content—along with six tepals enriched in anthocyanins and flavonoids, and three stamens, also referred to as anthers or styles. The stigmas are the botanical source of the saffron spice and must be carefully harvested and subjected to specific drying protocols. The method of drying significantly influences the quality and stability of the final product [22,23,24].

The major bioactive constituents of saffron stigmas include picrocrocin, crocins, and safranal. Crocins are the glycosylated esters of crocetin, a C_20_-dicarboxylic carotenoid derivative. Picrocrocin is a monoterpene glycoside responsible for saffron’s characteristic bitter taste, whereas safranal, a volatile monoterpenoid aldehyde, contributes to its distinct aroma. These compounds have been extensively studied using a range of analytical methods, resulting in the identification of various isomeric forms depending on the resolution and sensitivity of the applied techniques [24].

Commercial standards for saffron quality are established by the International Organization for Standardization (ISO), specifically in protocols ISO3632-1 [25] and ISO3632-2 [26]. These guidelines assess quality by measuring the absorbance of aqueous saffron extracts at specific wavelengths corresponding to each compound: crocins at 440 nm, picrocrocin at 257 nm, and safranal at 330 nm. However, these procedures exhibit certain limitations, most notably the poor aqueous solubility of safranal and the substantial sample mass required for reliable quantification [27].

Safranal (2,6,6-trimethyl-1,3-cyclohexadien-1-carboxaldehyde) arises primarily from the degradation of picrocrocin. This transformation occurs via enzymatic hydrolysis—typically involving glucosidases—or through acid-mediated processes, both of which can be triggered by heat. Besides safranal, several other volatile compounds contribute to the aroma of saffron, including isophorone (3,5,5-trimethyl-2-cyclohexen-1-one), its isomer (3,5,5-trimethyl-3-cyclohexen-1-one), 4-hydroxy-2,6,6-trimethyl-1-cyclohexene-1-carboxaldehyde, 2,6,6-trimethyl-2-cyclohexene-1,4-dione, and 2,6,6-trimethyl-1,4-cyclohexadiene-1-carboxaldehyde—an isomer of safranal. These aromatic constituents are likely formed through the oxidative cleavage of carotenoids under thermal and oxidative conditions [27].

Multiple safranal isomers have been identified using gas chromatography–mass spectrometry (GC–MS), as reported by Tarantilis and Polissiou, suggesting a complex aroma profile modulated by post-harvest processing [28].

In a more recent study, D’Auria et al. employed solid-phase microextraction coupled with GC–MS (SPME-GC–MS) to analyze the volatile profiles of saffron samples sourced from three Italian regions (Campania, Sardinia, and Abruzzo) and from Iran [29]. Their analyses identified 18 previously unreported volatile components and highlighted region-specific variations in chemical composition, likely attributable to differences in agronomic practices and environmental conditions.

Picrocrocin, the second most abundant apocarotenoid in saffron, is chemically described as 4-glucopyranosyloxy-2,6,6-trimethyl-1-cyclohexene-1-carboxaldehyde. It is biosynthesized via the enzymatic oxidative cleavage of zeaxanthin, mediated by carotenoid-cleaving enzymes, leading to the generation of crocetin dialdehyde and picrocrocin.

The pigment properties of saffron are primarily due to crocins, which are water-soluble carotenoid derivatives. The foundational work by Kuhn and L’Orsa first identified the principal pigment as the digentiobiosyl ester of crocetin [30]. Subsequent research expanded the catalog of crocetin glycosyl esters, identifying mono- and diglucosyl derivatives, monogentiobiosyl esters, and mixed glucosyl-gentiobiosyl esters [31]. Spectroscopic analyses, including UV/Vis, NMR, and elemental analysis, have facilitated the characterization of additional structural isomers, such as 13Z-crocin (see for reference Speranza et al. [32]). In recent decades, advancements in analytical sensitivity have allowed the identification of a wider range of glycosylated crocins, including triglucoside and neapolitanoside derivatives, adding to the structural complexity of saffron carotenoids [33,34,35]. A total of 16 distinct crocetin esters have been reported, some of which have also been detected in *Gardenia jasminoides * Ellis fruits [36].

In summary, the complex metabolite profile of saffron, characterized by a range of glycosylated apocarotenoids and volatile aromatic compounds, offers a rich foundation for exploring its potential therapeutic roles, particularly in neuroprotection. Understanding the interplay between botanical source, post-harvest treatment, chemical composition, and analytical methodology is critical for harnessing the pharmacological benefits of this valuable plant.

A detailed metabolomic analysis of the stigma—the most pharmacologically relevant part of the flower—has proven essential for understanding the relationship between its chemical composition and biological activity.

The stigmas of *C. sativus* contain a complex array of secondary metabolites, among which the apocarotenoids crocins, picrocrocin, and safranal are most significant. According to Sánchez et al. and Assimiadis et al., the most abundant crocetin ester is trans-crocetin di-(β-D-gentiobiosyl) ester (T1), which alone comprises over 60% of total crocin content in aqueous extracts [37,38]. Following this, the next most prevalent compounds are trans-crocetin (β-D-gentiobiosyl)-(β-D-glucosyl) ester (T2), cis-crocetin di-(β-D-gentiobiosyl) ester (C1), and cis-crocetin (β-D-gentiobiosyl)-(β-D-glucosyl) ester (C2). Collectively, the three major crocins (T1, T2, C1) represent more than 95% of the total crocetin ester pool in stigmas.

The proportion of these compounds can vary significantly depending on geographical origin, cultivation practices, and post-harvest processing. Generally, crocins constitute approximately 10% of the dry weight of saffron, while picrocrocin accounts for about 4%. Safranal, a key contributor to the aroma profile, represents around 70% of the volatile fraction [39].

The neuroprotective potential of saffron has been linked to its chemical profile, especially the concentration of crocins. Experimental work combining metabolomic profiling and in vivo pharmacological testing has demonstrated that the efficacy of saffron in neuroprotection is dependent on the abundance of T1 and T2 crocins. The effective neuroprotective action is not observed when T1 is present at levels below 17 mg/g, and T2 below 8 mg/g. These findings served as the foundation for filing an international patent and led to the development of a saffron quality certification known as Repron^®^ [21].

The integration of spectroscopic and pharmacological data underscores the importance of chemical standardization in saffron and supports the use of certified quality indicators like Repron^®^ to ensure efficacy in therapeutic applications.

## 3. In Vitro and In Vivo Data

Saffron experiments started in an animal model of light-induced retinal degeneration, and the first question was related to the maintenance of morphology and function (cfr. Table 1). The original idea was that saffron might act as an antioxidant, but it was immediately suggested by FGF results and confirmed by microarray experiments that the ways of action are more complex. Saffron treatment was tested in animal models and cell culture to elucidate possible ways of action, and it was found that many ways are activated, going from the direct control of membrane channels [13,21,40,41,42] to the reduced release of cytokines from microglia, maintenance of extracellular matrix morphology, and reduced protein degradation [20,43]. The results appear to be a positive control of neuronal death and neuroinflammation and, consequently, of function.

Microarray data [44] have provided evidence that saffron is able to modulate the activity of many genes and microRNAs (miRNA) involved in gene transcription [45]. Here, miR-124 expression is inversely correlated to chemokine (C-C motif) ligand 2 (CCL2) expression, and CCL2 is strongly modulated by saffron treatment [44]. Recently, an interesting review (Guo et al.) reported data on the link between CCL2 and its receptor CCR2 [46]. CCL2 is primarily produced by microglia and astrocytes under pathological conditions, while CCR2 is expressed by neurons, astrocytes, and infiltrating leukocytes but not by resident microglia, supporting the idea and explaining recent results on the relevant role played by saffron in modulating the immune system. Interestingly, even choroidal neovascularization depends on the axis CCL2-CCR2 [47]. Saffron treatment can cope with retinal neovascularization due to diabetic retinopathy (clinical observation), and probably this is why patients with dry AMD and an early starting of saffron treatment might never progress to the wet form. This possibility is also suggested from recent data, showing the relevant role of microglia in the control of retinal vascularization [48].

Saffron components appear to be able to modulate many cellular pathways, suggesting complex and coordinated ways of action. Based on these observations, it becomes clear that the saffron does not act as a simple antioxidant, supporting the hypothesis of an involvement of very different ways of action, going from antioxidant activity to direct control of gene expression. The obtained results showed good maintenance of both morphology and function. Animals exposed to high-intensity light present a destabilizing area in the dorsal retina, a few millimeters dorsal to the papilla, the so-called “hot spot” [49]. The degeneration induced by light starts in that location and progressively extends in adjacent retinal areas [50]. Saffron not only reduces photoreceptor death [14,51] but stabilizes the morphology of this area by reducing “rosettes” formation and inflammatory reactions [20]. The presence of fibroblast growth factor 2 (FGF2), relevant in the outer nuclear layer (ONL) after light exposure, is reduced by saffron pre-treatment. Interestingly, in microarray experiments [44], the FGF gene, up-regulated by light, is only mildly modulated by saffron, but the protein is absent, as it was proved by Western blot analysis and immune labeling, so we can advance the hypothesis that saffron components might regulate gene transcription products as suggested by the great number of ncRNA modulated by saffron. Retinal function was tested by recording ERG responses to light flashes of increasing intensity before and a week after light induced damage, both a- and b-waves were analyzed. Retinal response to light is partially preserved for both a- and b-waves, with a reduction in sensitivity, which is significantly different in treated vs. controls.

Saffron and its components have been tested in animal models with neurodegenerative diseases [18,52,53], retinal degeneration [11,14,21,41,43,51,54,55,56,57], and in human subjects with Alzheimer’s and depression [58,59,60,61]. It is interesting to note that in some animal models where crocetin was used [54,55] the effects were obtained with doses of 100 mg/Kg/day, and with safranal treatment, the dose was 400 mg/kg/day [57]; similarly, when a saffron extract was tested, the amount was over 50 mg/kg/day (up to 250 mg/kg/day in Hosseinzadeh et al., 2012) [52], while in our experiments [11,14,21,41,43,44,51,56] we used 1 mg/kg/day (5 mg in some experiments) of saffron where crocins represents about 4%. Even the ways of administration can vary from oral to intraperitoneal and intravenous injection; nevertheless, the outcome always provided positive results. Interestingly, in patients, the dose used was 30 mg/day; we used 20 mg/day. Some discrepancy in results can be due to the chemical composition of saffron, which in our experiments is strictly controlled, and the ratio among components corresponds to saffron Repron^®^, linked to a patent. This might explain the difference with clinical trials, where saffron treatment provided positive results but less efficiency [62]. The chemical composition is critical to obtain positive and reliable results, and it must be tested for each batch of saffron used to prepare pills.

**Table 1:** In this table, preclinical studies regarding the use of saffron alone or compared (or combined) with other compounds are described in in vivo and in vitro models of diseases.

Year	Pathology Model	Techniques	Supplementation	Main Outcomes	Reference
2016	661W, HEK293 P2 × 7R	Viability assay	Saffron at a concentration of 5 mg/mL	Saffron positively modulated viability in cell lines expressing P2 × 7 purinergic receptor, which has a role in pathobiology of RP and AMD	Corso L. et al. [40]
2019	ARPE-19	Proliferation and migration assays, flow cytometric viability assay, and quantification of apoptosis proteins	Crocetin at a concentration of 100–400 µM	Inhibition of migration and proliferation of RPE cells induced by PDGF modulating Bcl-2 family regulators and PI3K/Akt, ERK, p38, and JNK.	He Zhang et al. [63]
2023	ARPE-19	Cell viability assay and morphological markers of apoptosis	Hydroponical saffron and Repron at a concentration of 40 µg/mL	Increase in cell survival and protection from apoptosis	Di Paolo M et al. [64]
2024	661W	Viability and mitochondrial assays, study of mechanism of ferroptosis and pyroptosis	Crocin at a concentration of 200 µM	Protection from all-trans retinal accumuli damage, suppression of marker cell ferroptosis and pyroptosis	Bo Yang et al. [65]
2025	ARPE-19	Viability assays, evaluation of oxidative stress and inflammatory status	Saffron (at a concentration of 40 µg/mL) alone or combined with Elderberry and Melilotus officinalis	Protection from oxidative stress by saffron alone/combined with Elderberry and Melilotus Officinalis. A combinative effect was exerted by the mix in activating the NRF2 pathway	Puddu A et al. [66]
2025	ARPE-19	MitoLight staining, ZO-1 expression, TUNEL assay	Repron^®^ saffron extract (40 µg/mL), hydroponic saffron extract (40 µg/mL), whole-saffron-flower extract (150 µg/mL), and saffron tepal extract (150 µg/mL)	Different drugs obtained from the same plant could be useful to address several aspects of the pathology: the waste extract for the oxidative stress and inflammation, the tepal extract for acrolein levels	Galante A et al. [67]
2006	Primary retinal cell cultures, primate and bovine retinas	TUNEL assay	Crocin (EC50 = 30 microM)	Photoreceptor protection against light damage: concentration-dependent effect	Laabich A et al. [68]
2008	Sprague–Dawley rats	TUNEL viability assay FGF2 quantification and localization, fERG	Saffron extract (*Crocus sativus* L.) 1 mg/kg	ONL morphology preserved Photoreceptors’ function preserved only in the saffron	Maccarone R et al., [14]
2010	Albino rats	ncRNA	Saffron 1 mg/kg/day	Positive modulation of ncRNA	Natoli R. et al. [44]
2011	RGC-5, mice	Intracellular oxidation ROS, ER stress-related proteins, disruption of mitochondrial membrane potential, and caspases activation, ERG, TUNEL assay	Crocetin in vitro 3 microM, in vivo 100 mg/kg	Protection from tunicamycin and H_2_O_2_ damage in vitro, reduction in light damage in vivo	Yamauchi M et al., [54]
2012	P23H rats	Viability assays, ERG, capillary network	Safranal 400 mg/kg twice a week	It reduced the degeneration of photoreceptors, preserved function and avoided the disruption of capillary network.	Fernández-Sánchez L. et al. [57]
2012	Mice	TUNEL assay, caspase-3/7 and calpain activity, ERG	Crocetin at 100 mg/kg p.o	Mitigation of excitotoxicity effect of an IVT of NMDA in murine retina	Yuta Ohno et al. [55]
2013	Sprague–Dawley rats	TUNEL assay, PI3K/Akt quantification	Crocin 50 mg/kg p.o	Protection of the ganglion cells from ischaemia/reperfusion damage, activating the pathway PI3K/Akt. The effect is reversed with an IVT inhibitor of PI3K	Yun Qi et al. [69]
2013	Parkinson’s disease mouse model	TH+ cell count	Saffron 0.01% *w*/*v*	Saffron was able to mitigate the influence of MTPT on dopaminergic neurons in SNc and retina.	Sivaraman Purushothuman et al. [53]
2013	Sprague–Dawley rats	TUNEL assay, thickness of ONL, GFAP expression	Saffron 1 mg/kg	Saffron or PBM preconditioning could be of use in protecting from light damage	Di Marco F. et al. [51]
2013	Mice	TUNEL assay, 8-OHdG, phosphorylation of MAPK, ERK, JNK, p38, NF-kB, c-Jun, ERG	Crocetin 20 mg/kg p.o	Reduction in the oxidative stress in the mouse model of ischemia/reperfusion	Ishizuka F. et al. [70]
2014	Sprague–Dawley rats	TUNEL assay, ONL thickness, ERG	Saffron 1 mg/kg/day, Photobiomodulation	The two neuroprotective approaches do not exert a combined effect on light-induced death in a rat model.	Di Marco F et al. [71]
2015	Rats	Viability assays, evaluation of oxidative stress (glutathione levels, total superoxide dismutase and ROS), and evaluation of p-ERK)	Crocin 50 mg/kg	Protection of ganglion cells from ischaemia/reperfusion damage decreases caspase-3 and p-ERK, increasing the levels of GSH and T-SOD.	Chen Li et al. [72]
2016	Sprague–Dawley rats	Quantification of protein and RNA of ECS pathway, viability assays, and ERG	Saffron 5 mg/kg/die	Reduction in the expression of CB1 and CB2, increased by photo-induced damage.	Maccarrone R. et al. [41]
2018	ApoE^−/−^ mice	Lipidemic profile, glucose, C-reactive protein, total oxidative capacity, retinal thickness	Saffron 25 mg/kg/die	Saffron supplementation per os 25 mg/kg/die could help in improving oxidative profile in blood and preservation of retinal thickness.	K Doumouchtsis et al. [73]
2019	Sprague–Dawley rats	(1) SDS-PAGE analysis; (2) Western blotting; (3) enzyme activity assay; (4) Immunolabelling	Saffron 1 mg/kg/die p.o	1 mg/kg/day p.o. of saffron was administered. Enzymatic activity of MMP-3 is reduced	Di Marco S et al. [20]
2019	Mice	inner retina thickness, MMP-9, TNF alpha and occludine expression	Crocetin 100 mg/kg	Reduction in the retinal edema and the expression of MMP-9 and TNF alpha, reversing the reduction in occludine, in a murine model of retinal vein occlusion.	Nitta K et al. [74]
2019	Glaucoma mice	viability assays, morphology assays of microglia activation, and P2RY12 expression	Saffron (3% of crocin)	Treatment reduced markers of neuroinflammation and partially reversed The hypoexpression of P2Ry12	Jose A Fernández-Albarral et al. [75]
2020	Light-induced damage in mice, 661W, HEK293 P2X7R	Morphological assays, viability assays	Saffron 25 μg/mL	Trans-crocetin bis (β-d-gentiobiosyl) ester is the most neuroprotective compound among the crocetins in saffron	Anna Maggi et al. [21]
2020	Wistar rats	activity of SOD, GPx, Cat, MDA	Saffron 60 mg/kg	Saffron has a protective potential in retinas of diabetic rats	Skourtis G. et al. [76]
2021	Glaucoma mice	activity of SOD, GPx, Cat, MDA	Saffron 60 mg/kg	Saffron mitigates the increase in inflammatory chemokines in laser-induced OHT.	José A Fernández-Albarral et al. [77]
2021	Mice, RCS rats, RCS-rdy rats, Fischer rats	TUNEL assay, ERG, IHC and WB for Sirt1, Sod1	Naringenin, saffron 1 mg/kg/day	Saffron exerts its neuroprotective effect in a setting ruled by lipid peroxidation, while naringenin can be protective in the first stage of redox stress	Piano I et al. [11]
2021	Albino rats	ONL thickness, neuroinflammatory markers for microglia	Saffron 1 mg/kg/day	A combinative effect of PBM and saffron supplementation was observed only in the early neuroinflammation.	Di Paolo M. [78]
2023	Mouse model of LPS-induced neuroinflammation	Novel Object Recognition, ERG	Saffron 10 mg/kg/day	Chronic treatment with saffron Repron can reduce neuroinflammation, slowing cognitive and visual impairment	Di Paolo M. [79]
2023	Albino rats	MDA, IL6, TNF alpha, TEM, IHC for caspase 3, COX-2, and GFAP	Saffron 80 mg/kg/day	Saffron can improve retinal redox status, inflammation, and histological changes, protecting from the effects of Sofosbuvir	Elseady Walaa et al. [80]
2023	Glaucoma rats	IHC staining for TNF-α, IL-1β, and IL-6, and BDNF in primary visual cortex. WB for PI3K, Akt, and NF-κB in the retina	Crocetin 55.38 mg/kg, 27.69 mg/kg, and 13.85 mg/kg	Crocetin shows neuroprotective effects, lowering the quantity of inflammatory chemokines and increasing the concentration of BDNF	Li Q et al. [81]
2024	1. ARPE-19 2. Umbilical vein endothelial cells 3. Mouse CNV model	viability and migration assays, expression of VEGF, HIF-1alpha, ZO-1, TNF alpha, IL-1B, IL6CNV evaluation	Crocetin at concentrations of 100 and 200 μM	Inhibition of VEGF-induced cell proliferation, reduction in choroidal sprouting and CNV size, and reduction in markers of hypoxic cell injuries	Wang C et al. [82]
2025	Rd10 mice	ERG, immunohistochemistry, Western blot, RT-qPCR, Behaviour	Saffron 10 mg/kg/day	Prolonged saffron treatment is effective in slowing long-term damage in rd10 mice in neural retina through acquired resilience	Corsi F et al. [83]

*ARPE-19*: Arising human RPE cell line, *ERG*: elettroretinogram, *IHC*: Immunohistochemistry, *LPS*: lipopolysaccharide, *Mitolight*: measurement of mitochondrial membrane potential, *PBM*: photobiomodulation, *P23H rats*: slow rod degeneration rats, *RCS rats*: Royal College of Surgeon rats, *rd10 mice*: AR Retinitis Pigmentosa mouse model, *RGC5*: retinal ganglion cell-line, *WB*: *Western blot*, *TH*: tyrosine hydroxylase, *TUNEL*: Terminal deoxynucleotidyl transferase dUTP Nick-End Labeling, *ZO-1*: Zona Occludens-1, *661W*: murine cone photoreceptor-derived cell line.

## 4. Saffron Pharmacokinetics

A key unresolved question concerns which metabolites reach target tissues and remain bioactive after oral administration. Numerous studies have demonstrated a variety of pharmacological actions for crocetin and crocins, the supposedly active saffron components, e.g., enhancement of oxygen diffusivity [17], increment of eye blood flow [84], inhibition of tumor cell proliferation [85,86,87], and protective effects against a variety of diseases [51]. However, in contrast to these investigations aimed to evaluate biological activities, there are few studies on the absorption and metabolism of crocetin and crocins. Asai et al. [88] investigated the absorption of orally administered crocetin and crocins into the blood circulation in mice. Their results suggested that crocins are hydrolyzed to crocetin before or during absorption and then undergo the metabolic pathway of crocetin. Hence, according to these data, orally ingested crocins could not act as bioactive molecules by themselves “in vivo” except in the gastrointestinal tract. The AA proposed a model of the metabolic pathway of orally administered crocetin and crocins in mice (Figure 6 in ref. [88]). Orally administered crocins would be hydrolyzed to crocetin before being incorporated into the blood circulation. Thereafter, crocetin would be partly metabolized to mono- and diglucuronide conjugates. Crocetin is likely to be metabolized to glucuronide conjugates both in the intestinal mucosa and in the liver of mice. The rapid absorption and glucuronidation of crocetin indicate that the metabolic fate of crocetin is quite different from that of common C40 carotenoids. These analyses have been extended to rats by Xi et al. [89], and their results confirm that orally administered crocins remained undetectable in plasma, further indicating that the intact form of crocin was not absorbed from the intestinal tract. In addition, plasma crocetin concentrations do not tend to accumulate with repeated oral doses of crocin, and the intestinal tract serves as an important site for crocin hydrolysis. At present, these are the only available data on the metabolism of ingested crocins and crocetins. In humans, Umigai et al. [90] clearly demonstrated that crocetin was more rapidly absorbed than the other carotenoids and was subsequently eliminated from human plasma within a maximum of 8 h. Considering the neuroprotective effects of oral saffron supplementation both in animal models and AMD patients, we examined whether it was possible to find metabolites leading to saffron components in different tissues. Specifically, we used an animal model with induced photoreceptors degeneration (see Bisti et al.; Maggi et al. [21,56]) pre-treated with dietary saffron with a dose five times higher (5 mg/kg/7 d) to verify whether saffron related metabolites were found in the stressed retina, blood, urine, kidney, liver and cerebral cortex. As a control, we used treated animals with no degeneration. In addition, we tested blood and urine from healthy voluntaries and AMD patients treated daily with saffron for long time (over a year).

We analysed altogether 15 animals with saffron treatment and LD, and five controls (treatment without damage). All animals were sacrificed in the morning under comparable conditions, saffron was supplemented in the drinking water, and the daily dose was diluted in a volume that was always finished in one day. In agreement with previous reports, we found crocetin in all blood samples, but no traces of metabolites related to saffron were ever found in any other tissue examined except for degenerating retinae. In seven retinas out of 15 animals, we found crocins, although not in a relevant amount, and we advance the hypothesis that crocins are resynthesized from circulating crocetin, which reaches the retina following damage to the blood-brain barrier. To support this hypothesis, we have evidence that we did not find any metabolites in healthy retina and the central nervous system, nor crocin nor crocetin. We also examined blood and urine from two AMD patients under saffron treatment for over a year and three healthy volunteers after two weeks of treatment with the same daily dose (20 mg/per day). Samples were taken two hours after intake of the morning saffron pill. Interestingly, while in volunteers it was always possible to determine the presence of crocetin in both blood and urine, the same procedure was often unsuccessful with patients, as if all the metabolites were immediately absorbed and used. Kanakis et al. [91], in their study on the interaction of crocetin with human serum albumin, demonstrated that the binding of crocetin shows a weak ligand–protein interaction. Since the less bound substance to plasma protein tends to easily distribute to tissues of the body, crocetin is expected to be well distributed in the system. These preliminary results possibly explain why the positive effects on visual function revert if the intake of saffron is interrupted.

Safety data are favorable. Such data have been recently reported by Ayatollahi et al. [92]. The study was a double-blind, placebo-controlled study consisting of a 1-week treatment with 200 mg and 400 mg saffron tablets. Sixty healthy volunteers (age range 20–50 years) were selected for the study. The administration of saffron tablets (200 or 400 mg/day for 7 days) did not induce any significant modification in the coagulation or anticoagulation system. The dose used in AMD patients is twenty times lower, and it must be considered that metabolites seem to be immediately used. Altogether, our and the literature-reported results provide evidence of a treatment with a high degree of safety and no side effects, at least at the dose used in humans.

## 5. Clinical Trials

A phase I/II study in early AMD (clinicaltrials.gov NCT00951288) randomized patients to oral saffron (20 mg/day) or placebo for three months, with crossover for an additional three months. Focal electroretinograms (fERGs) and clinical findings were recorded at baseline and after three months of either saffron or placebo supplementation. fERGs were recorded in response to a sinusoidally modulated (41 Hz) uniform field presented to the macular region (18°) at different modulations between 16.5% and 93.5%. Main outcome measures were fERG amplitude (in μV), phase (in degrees), and modulation thresholds.

After saffron, patients’ fERGs increased in amplitude, compared either to baseline values or to values found after placebo supplementation (mean change after saffron: 0.25 log μV, mean change after placebo: −0.003 log μV, *p* < 0.01). fERG thresholds decreased after saffron, but not placebo supplementation compared to baseline (mean change after saffron: −0.26 log units; mean change after placebo: 0.0003 log units). The results indicate that short-term dietary saffron supplementation improves retinal flicker sensitivity in early AMD.

Subsequent AMD trials replicated functional benefits [62,93,94,95], albeit with smaller effect sizes, likely reflecting heterogeneity in chemical composition; our studies used composition-defined, patented material. These findings suggest that saffron engages the AMD pathophysiology beyond simple antioxidant mechanisms.

To evaluate whether the observed functional benefits from saffron supplementation may extend over a longer follow-up duration, a longitudinal, interventional, open-label study in an outpatient ophthalmology setting was conducted. Twenty-nine early AMD patients (age range: 55–85 years) with a baseline visual acuity of >0.3 were enrolled. They had dietary saffron supplementation (20 mg/day) over an average period of treatment of 14 (±2) months. Clinical examination and focal electroretinogram (fERG)-derived macular (18°) flicker sensitivity estimates were collected every three months over a follow-up of 14 (±2) months [15]. Retinal sensitivity, the reciprocal value of the estimated fERG amplitude threshold, was the main outcome measure. After three months of supplementation, mean fERG sensitivity improved by 0.3 log units compared to baseline values (*p* < 0.01), and mean visual acuity improved by two Snellen lines compared to baseline values (0.75 to 0.9, *p* < 0.01). These changes remained stable over the follow-up period. These results indicate that in early AMD, saffron supplementation induces macular function improvements from baseline that are extended over a long-term follow-up.

To determine whether the functional effects of dietary supplementation with saffron are influenced by major risk genotypes such as complement factor H (*CFH*) and age-related maculopathy susceptibility 2 (*ARMS2*), a long-term assessment was conducted in early AMD patients who were preliminarily assessed for these gene polymorphisms [9]. Thirty-three early AMD patients, genotyped for *CFH* (rs1061170) and *ARMS2* (rs10490924) polymorphisms and receiving saffron oral supplementation (20 mg/day) over an average period of treatment of 11 months (range, 6–12), were longitudinally evaluated by clinical examination and focal electroretinogram (fERG)-derived macular (18°) flicker sensitivity estimate. fERG amplitude and macular sensitivity, the reciprocal value of the estimated fERG amplitude threshold, were the main outcome measures. After three months of supplementation, mean fERG amplitude and fERG sensitivity improved significantly when compared to baseline values (*p* < 0.01). These changes were stable throughout the follow-up period. No significant differences in clinical and fERG improvements were observed across different *CFH* or *ARMS2* genotypes [9]. These results indicate that the functional effect of saffron supplementation in individual AMD patients is not related to the major risk genotypes of disease. In conclusion, saffron treatment appears able to cope with the degenerative progression of AMD. Although the ways of action are still under investigation, it seems reasonable to conclude that saffron components can reduce photoreceptor death, acting at different levels in a synergistic way and probably increasing tissue resilience [96,97]. Looking at the chemistry of various molecules, a direct antioxidant activity might be hypothesized, thus reducing the oxidative stress associated with the initiation and progression of the disease. Recent results seem to suggest, based on data present in the literature and our own data, that the ways of actions are not limited to antioxidant activity but to complex and integrated actions able to cope with dysmetabolic events like apoptosis and neuroinflammation. Interestingly, all the chemical components appear to contribute to the efficacy of the treatment, but in a clearly defined ratio. Altogether, all data obtained confirms the driving idea that testing natural compounds, which include many molecules, must be done in parallel by different disciplines to optimize the results and offer patients a well-characterized and most effective treatment. An extensive list of trials involving the use of saffron is available in Table 2.

## 6. Future Directions and Conclusions

**Figure 1:**
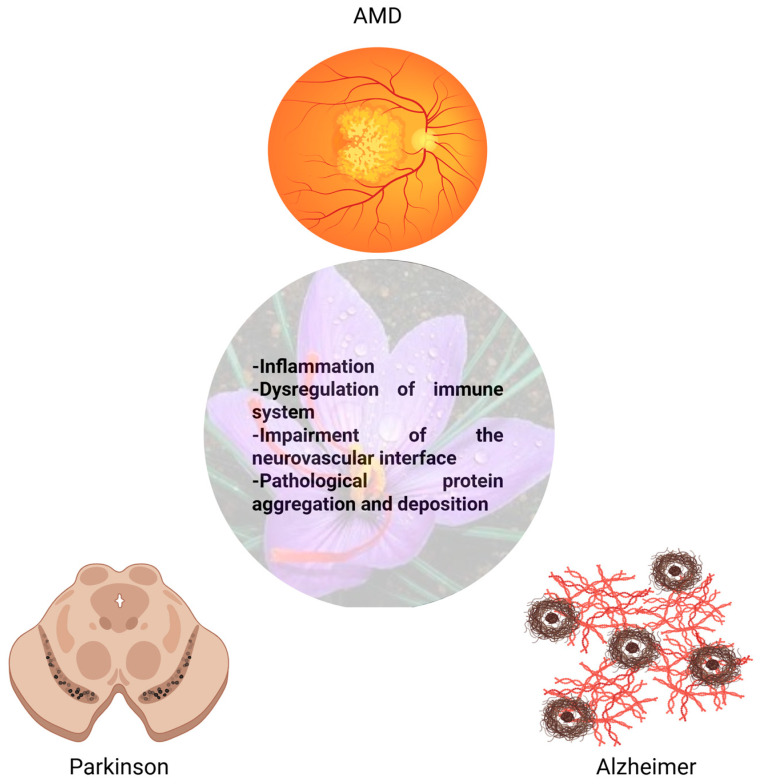
Neurodegenerative diseases of the eye and the brain, and saffron target. Created in BioRender. Mastromartino, R. (2026) https://BioRender.com/0j8gdvf (accessed on 20 March 2026).

Saffron is a collection of compounds proven to be effective in decelerating neurodegenerative processes in the brain. Its effects are also measured in the retinal tissue, where it slows ERG response deterioration in AMD patients. Its pleiotropic effects could be seen in the key of hormesis and acquired resiliency in terms of slowing degenerative processes affecting the neural tissue, since the retina is, from an embryological point of view, a light-explorable portion of the diencephalon [101]. Its effects on the retina could be attributable to an interesting characteristic of the examined retinal disorders: AMD shares pathophysiological features with Parkinson’s and Alzheimer’s diseases. They can be summarized as follows (see Figure 1) [102]:

- Common pathways of inflammation, oxidative stress and membrane organelles homeostasis [103].
- Dysregulated innate and adaptive immune responses [104].
- Dysfunction at the neurovascular interface [105].
- Pathological protein aggregation and deposition [106].

In this contest, saffron acts as a neuroprotectant, regulating the neuroimmune axis of CCL2-CCR2 [44,46] and protecting the neurovascular barrier through microglia [47]. Moreover, in vitro studies showed its anti-aggregating properties, inhibiting deposition of tau protein filaments [107].

From a clinical perspective, it is known that symptoms and instrumental findings could appear either in CNS degenerative disorders or in AMD, such as deterioration of visual acuity, contrast sensitivity, and cognitive measurements [107,108,109]. In this framework, saffron has its own efficacy as one of the most effective groups of molecules tested and has a primary role in alerting to the shared perspectives of similar supplements in retinal and CNS disorders. However, readers should be aware that such a supplement with pleiotropic effects should be used not only as a means to delay pathology, but also as a model of treatment to elucidate the physiopathology of the neurodegenerative diseases of the eye and the brain.

## Figures and Tables

**Table 2:** In this table, clinical trials involving the use of saffron are listed, with the main outcomes being described.

Year	Pathology Model	Study Design	Supplementation	Main Outcomes	Reference
2010	Early AMD	Randomized clinical trial, placebo-controlled, crossover	20 mg/die saffron for 3 months	Improvement of focal ERG amplitude	Falsini B. et al. [15]
2012	Early AMD	Clinical trial	20 mg/die of saffron for 3 months	Improvement of focal ERG amplitude and derived macular sensitivity	Piccardi M. et al. [16]
2013	AMD	Trial	20 mg/die of saffron	Improved fERG amplitude and derived macular sensitivity, not conditioned by *CFH* or *AMRS2* risk polymorphisms	Marangoni D. et al. [9]
2018	Diabetic macular rdema	Randomized, double-blinded, placebo-controlled, clinical trial	5 mg or 15 mg crocin tablets per day for 3 months	BCVA, CMT, HbA1c. Improvement in all outcomes only in the 15 mg saffron group	Samaneh Sepahi et al. [98]
2019	Stargardt disease	Randomized, double-blinded, placebo-controlled trial, crossover	20 mg/die of saffron for 6 months	focal ERG, unchanged after saffron supplementation.	Piccardi M. et al. [99]
2019	Moderate AMD	Randomized, double-blinded, placebo-controlled clinical trial, crossover	20 mg/die for 3 months	BCVA, mfERG. Improvement in all outcomes.	Geoffrey K Broadhead et al. [62]
2019	AMD	Longitudinal open-label study in two groups of patients: lutein/zeaxanthin and saffron-treated	lutein and zeaxanthin, saffron	Visual function remains stable in AMD patients treated with saffron	Di Marco S et al. [20]
2024	Neovascular AMD	Randomized clinical trial, with a healthy control group	lutein and zeaxanthin, saffron	Micronutrient supplementation may help restore the gut–retina axis	Baldi S et al. [100]
2024	Mild/moderate AMD	Open-label, extension trial	20 mg/die of saffron	Saffron supplementation modestly improved mfERG measures, especially in the central rings	GK Broadhead et al. [94]

*AMD*: Age-related Macular Degeneration, *BCVA*: best-corrected visual acuity, *CMT*: central macular thickness, *mfERG*: multi-focal electroretinogram.

## Data Availability

No new data were created or analyzed in this study. Data sharing is not applicable to this article.
